# History of OPO- or Organo-Therapy1President’s annual address to the Buffalo Academy of Medicine, June, 1899.

**Published:** 1899-09

**Authors:** Roswell Park

**Affiliations:** Buffalo, N. Y.; Professor of surgery, Medical Department, University of Buffalo


### Selections.

### HISTORY OF OPO- OR ORGANO-THERAPY.[1]

By ROSWELL PARK, A. M., M. D., of Buffalo, N. Y.
Professor of surgery, Medical Department, University of Buffalo.

[From St. Louis *Courier of Medicine* for August.]

HOW little new there is under the sun has been subject of frequent comment by students from time immemorial. That this may apply equally well to the general subject of opo-therapy or organo-therapy, can easily be made to appear. Those of us who are now using the various animal extracts, having begun to use them because we were advised so to do, have little idea of the real history of the subject or of the method, either by which they came into use or by which they have come to be given in their present form. And so it has occurred to me that an historical review of this subject would not be without its present interest and importance, and for this purpose I have availed myself of the studies published by Hopf, and published in the current issues of "Janus" (*Zur Geschichte der Organo-Therapie.*)

Although one can scarcely trace the use of our present elegant pharmaceutical preparations back to the crude use of various parts of animal bodies from prehistoric time, it is hard to say just how largely present methods are due to prevalent superstitions and popular notions or to definite laboratory and experimental investigations. It will probably appear to you that they are due to both.

Of the four ancient Egyptian Papyri which concern the practice of medicine, the so-called *Papyrus Brugsch* is the principal one which has been carefully gone over, and from which we are as yet repaired to draw conclusions. This, with the *Papyrus Ebers*, furnishes us abundant illustration of the reliance placed in those ancient times upon, for instance, the viscera of animals and the excrement of the gazelle as powerful remedies for that condition of the eye which we must suppose to have been the Egyptian ophthalmia. Various animal secretions and excretions were recommended in this work, particularly for maladies of the eyes. Among others, it was advised to take a human brain which was to be divided into two halves. One half was to be mixed with honey and rubbed upon the eyes in the evening; the other half was to be dried, pulverised and

1. President's annual address to the Buffalo Academy of Medicine, June, 1899.

used upon the eyes in the morning. Blood of various animals was also supposed to be an effective medium for chemosis or edema of the conjunctiva, and the milk of a nursing woman was then, as often now, considered most effective. Ox gall was considered a good remedy for worms, as it probably is today in certain instances.

One would, perhaps, expect of so deliberate and accurate a thinker as Hippocrates, that these medicinal phantasies of the Egyptians would scarcely find a place in his writings; but even this great master of antiquity lent himself to popular belief in this direction, and milk of a nursing woman was an important therapeutic medium in his estimation. Among the dogmatists who followed Hippocrates there were those who recommended the decoction made by cooking young puppies in wine as a means of curing sterility in women; while various insects, beetles especially, were advised as emmenagogues. In the time of Erasistratus the brain of a camel was considered a most excellent remedy in epilepsy, as were also the testicles of a wild boar. In Rome, in the time of the Emperor Trajan, Archigenes recommended very highly the flesh of the viper. But it was Pliny, especially the elder, who seemed to have most trust in this sort of medication, and he called attention to the fact that many of the Greek physicians seemed to have a great faith in the use of certain of the viscera or parts of the body, even including the tips of the finger nails, after they had been cut off. He alludes to the fact that some eminent men attach great value to the skull bones of a criminal, and that Antaios, especially, advised pills made out of the skull of an executed criminal for bite of a mad dog. And then Pliny goes on to say that we cannot emphasise sufficiently how much we owe to the Romans in that they put a stop to this practise of even murdering men in order to secure from their bodies something which could be used as a remedy, such as the skull bones or the brains of little children, etc. It was not quite so bad when the medical men changed their practise to such an extent as to substitute the liver of the mad dog and employ it as against his own bite, or to recommend the liver or the juice of the same drunk in wine, or gall, especially from the gall bladder of the bear or the wild boar, or the dried or powdered lung from the same animals. Also the heart was supposed to be efficient in epilepsy, especially when taken from the animal in the first or the second day of the new moon, and particularly if the animal were a black male ass.

The spleen has almost vied in alleged power with the liver, and Pliny particularly recommended the spleen removed from a living dog and, taken with milk, for affections of the same viscus. acknowledging, however, that the cow's spleen would probably answer as well. The stomach of the fowl cooked and taken in decoction was supposed to be good for pulmonary troubles as well as for intestinal affections. For kidney diseases the kidney of a hare or of the ass, taken with wine, was supposed to be peculiarly efficacious, as well as for stone in the bladder. In fact, we get here our forerunner of the isopathy of the last century, in that for bladder affections the bladder of the wild boar was recommended to males and of the wild sow for females. The testicle of a hippopotamus infused in water was recommended by Pliny as an effective agent against the bite of the serpent. So the female sexual organs of some of the animals were also recommended to women suffering from pelvic disorders peculiar to their sex, and for difficult labor the membrane in which a young goat came into the world was a sure relief, if taken in wine.

Blood, also, was supposed to have much the same virtue in those days as now, and Pliny regarded it as the most valuable of all these remedies from the animal kingdom. For instance, in dropsy, the blood of a goat was advised, while for pulmonary hemorrhages the blood of a young goat, drunk warm, with a equal quantity of vinegar, was a sure cure, as was also the blood of a wild ass for whooping-cough.

Entire animal bodies even were of reputed efficacy for certain affections. Thus, for the nocturnal incontinence of children it was recommended to give a boiled mouse, while the bodies of swallows, taken either fresh or burnt to ashes and drunk in water, were supposed to be good for headache and sore throat. Again, it was recommended to take twenty-one red flies in one potion for epilepsy. This is not so very widely different a practice from that of today, when the Spanish fly is still an accepted feature of our pharmacopeia. Against colic Pliny recommended the ashes of the hard portion of the hart's horn mixed with African snails, while snails alone were supposed to be good for cough and expectoration, especially when rubbed up raw in water. And so one might follow both the elder and the younger Pliny through an almost endless list of such measures as these, given in all seriousness and apparently with a firm faith in their efficacy. Later, under the Emperor Antonius Pius, Marcellus wrote a sort of poem upon the medicinal virtues to be secured among the fishes, and even the great Galen recommended kidneys of a certain fish as a positive aphrodisiac.

The Arabian physicians lent themselves to this same sort of belief in various forms, while the scholiasts of the Middle Ages did not depart from the teachings of Aristotle and Pliny. Gilbertus Anglicus (XIII. Century) earnestly recommended the flesh of the lion for fever and for stone in the bladder, as well as the blood of a young goat which had been previously fed upon certain herbs. Some people like Albertus Magnus, also during the XIII. Century, were outspoken in their faith in these remedies, and one in particular, Conrad, published a Book of Nature, which was little more than a repetition of the recommendations of Pliny. He advised the liver of the serpent in serpent bite and the flesh of the hart in fever, while lion flesh was good for chilling of the body. In asthmatic difficulties the flesh of the fox was to be eaten. Conrad's book had a large influence for two or three centuries, appearing in repeated editions. And even three hundred years later we find numerous disciples of his, especially in Germany, who were recommending all kinds of animal remedies for all kinds of disease. Even Paracelsus, reformer and reactionary as he was, could not quite get away from remedies of this kind.

In the organo-therapy of the Middle Ages, and in fact from the time of the Romans until the present century, the sexual organs of male animals have always occupied an important place in public esteem as means of rejuvenation and increase of vitality. When Brown-Sequard first introduced his testicular fluid, there obtained in it nothing of novelty, save the minute method of its preparation. In the New London Dispensatory, published by Dr. Salmon in 1684, it was stated that the external genitals of certain animals were good for sterility and various sexual weaknesses. Many different animals were used for this purpose. Especially was the testicle recommended when dried and drunk in wine. In this same connection Salmon recommended caviar as being especially nourishing and increasing virility.

These recommendations came with some force from a professional man. On the other hand, in 1702, there was published by Florinus a work with the title, The Wise and Intelligent Head of the Family. In this work were recommended various parts of nearly all the animals of Germany, as well as various portions of the human body. For instance, he advises the use of *cranium humanum preparatum*, made from the pulverised skull of criminals, for various affections, including constipation, although a much more effective agent for obstruction, according to Florinus, is the *oleum cranii humani*, which, however, must be made from the head of someone who has met a violent death. Florinus frequently lapsed into poetry, and became particularly poetic over the virtues of the deer, five different portions of whose anatomy were supposed to have marked effect in various diseases. Florinus did not esteem excrement so highly as a therapeutic material as did one of his successors, Paullini, who published a book upon the subject in 1714, which was republished one hundred and thirty years later. Scatology was in this work given the importance, almost, of a system, which had all the repulsiveness of isopathy, or even more. However, if one wishes to realise all that scatology means, he should consult the work on this subject by Bourke, one of our own army officers. These gentlemen also recommended the brain of the squirrel for faintness and vertigo, and it is stated that the Alpine hunters of that time gave to their pregnant wives meat of the squirrel in order that they might bring boys into the world, as well as that these boys might not suffer from dizziness or fall into the valleys.

Another book of no little interest appeared in Brabant in 1800. It was a book of approved and tried remedies and of Egyptian secrets, for man and beast. It was known usually as the "Book of Secrets," for short. Among other recommendations was this for dysentery, especially of the bloody form: That one should take the short rib from a criminal who had been hung, powder it and give this to the patient in a glass of wine or of vinegar.

And so one might go on and show how from the earliest days even to the present, mankind have believed in many things which the better sentiments of the educated condemn, and with which we today view with disgust.

I have alluded more than once to isopathy as a most discreditable and revolting offshoot from homeopathy. It is generally supposed that isopathic practises were long since discontinued, and yet I have today in my desk a price-list of a New York dealer in alleged homeopathic medicines, which are practically isopathic remedies, and which include, for instance, drugs and measures of the character so far mentioned in this paper. It certainly should shock the honest and sensitive homeopath even of today to see advertised for sale at one of his pharmacies such remedies as pus from a carbuncle, blood from a menstruating woman, and the like. Yet these things are actually mentioned in this price-list and can be bought at this moment in New York, as far as I know, since the dealer's address can still be found in the directory, or could until very recently.

So much as has been thus far said has reference purely to the crude and almost lawless method in which various parts of animal bodies have been employed in past centuries. It is introduced, however, merely to show that there was nothing new nor novel in the employment of any part of almost any animal body, but that it remained for modern scientists to study the action of these various substances, and show the way by which they could be occasionally employed.

And so it came about in the process of time that E. de Cyon presented a communication to the Paris Academy of Medicine, some years ago, upon the functions of the thyroid and upon the discovery therein of a certain compound in which iodine was plainly present, and which then, as now, bore the name of iodo-thyrine. According to Cyon it was the function of the thyroid to bring the iodine compounds in the blood into certain organic combinations, and in this way to remove from the nerve centers more or less dangerous and toxic substances. From experiment it appeared that iodine possessed paralysing activities upon the pneumogastric and cardiac centers, while the iodo-thyrine formed in the thyroid seemed to have activity in controlling the contraction of the heart and the circulation of the blood. This function of the thyroid was so far separated from that of the heart as to be manifested only through the influence of certain nerves. Inasmuch as the heart under the influence of iodo-thyrine produced a well-marked dilatation of the thyroid vessels, it appeared that the thyroid itself acted as a mechanism for the protection of the brain from sudden vascular changes, either by opening wider the thyroidal vessels or by increasing the amount of iodo-thyrine. Hence followed naturally the use of iodo-thyrine, particularly in cases of vascular and hyperemic goitre, as well again as in certain cases of thyroidal atrophy and cachexia strumipriva. In spite of all these theoretical deductions, there were but few practical applications of iodo-thyrine, at least few reported in literature, up to the beginning of 1897. About this time Grotz presented to the Vienna Dermatological Society a case of psoriasis which had been apparently healed in four weeks by the use of iodo-thyrine. In various places the remedy came into general and rapid use, and results were reported from numerous foreign clinics. Thus, Hanszel reported to the Vienna Clinical Society 220 cases of struma which had been treated in the outpatient department during 1896. About this time Merck put on the market tablets of thyroidin, whose use was followed by benefit, especially in the parenchymatous form of goitre, or even in the cystic form, but which seemed to be of no efficacy in the vascular form. While in many cases they were efficacious, a complete disappearance of the struma following their use has at no time been reported.

In exophthalmic goitre, also, thyroid preparations came to be generally tried, and even iodo-thyrine was used, although the indication for its employment was by no means very strong. Results were very contradictory, and even now one can form no positive judgment as to the effect of these preparations in such cases, every resort to them being in the nature of a judicious experiment. In 1896 also, Ewald summed up the matter before the congress of German physicians in Wiesbaden, when he said that the desirable results of thyroid medication seemed most conspicuous in myxedema, cretinism and like conditions of early life, whereas the result in skin diseases and goitre was often disappointing, sometimes even injurious. In spite of Ewald's warning, men used thyroid preparations unscientifically and unfortunately, as, for instance, even endeavoring with it to treat deafness which was due entirely to sclerotic processes. Nevertheless, in ichthyosis and perhaps certain allied forms of skin lesions, thyroid preparations seem to be indicated, perhaps because of their effect upon the bloodvessels of the skin. In the parenchymatous goitres, and sometimes even in tuberculosis of lymph nodes, the drug seems to have a beneficial effect. So also in reduction of obesity and in the internal treatment of the uterine myomata, although in the long run it would be best in these cases to limit its use to the inoperable cases. Gautier has found benefit also from thyroid preparations in delayed union after fractures, basing his first use of the drug upon certain laboratory results on the effect of the thyroid upon the growth of the bones.

Referring again, for a moment, to exophthalmic goitre, it would appear from numerous experimental studies that the disease itself might be ascribed, at least to some extent, to what has been spoken of as hyperthyroidism, and that when by employment of thyroid preparations an atrophy of the thyroid body can be produced, its peculiar products or secretions are thereby diminished, its vascularity decreased and benefit thus brought about. There is, therefore, this contrast between Basedow's disease and myxedema. A certain warning should be given, however, in all of these cases, based also upon careful experimental studies, to the effect that the prolonged use of thyroid preparations may produce a glycosuria of alimentary origin, and this makes it particularly wise to abstain from such preparations in diabetes.

Traczewski studied this subject in another way. He removed thyroids from animals and then fed them exclusively with animal food, and found that it had a very unfavorable effect upon them. He tried also the effect of the salts of phosphorus, particularly of sodium phosphate, upon these animals, and especially upon those in which he had left a portion of the thyroid, and found that these salts produced marked cachexia and finally atrophy of the remaining portion of the thyroid body. He found also the converse of this to be true, and thus we have a definite physiological and experimental basis for the employment of sodium phosphate in Basedow's disease. This tallies exceedingly well with the known clinical advantages of its exhibition in this malady.

A curiosity pertaining to this line of study is the attitude taken by Monk, who has been one of the most ardent students of the whole problem. In spite of all that laboratory investigation and clinical experience have shown in regard to the functions of the thyroid, and the effect of the thyroidal preparations demonstrated experimentally, he denies the presence of toxic substances in the body after removal of the thyroid, as well as that the peculiar symptoms due to its removal can be in any way combated from without. He claims that half of the animals from whom he has removed the thyroid show no unpleasant effects, and that myxedema is one of the rarest of post-operative phenomena. How completely he differs in these respects from some of his colleagues it is not necessary here to emphasize.

The next noteworthy effort in this direction was the now well-known, and then widely published attempt of Brown-Sequard, to extract from the testicles of animals a material which should so far influence nutrition and affect vitality as to rejuvenate the senile. There probably is no doubt as to the earnestness of the originator of this method, but the sensational aspects of the whole affair were so pronounced as to rob it of most of its value and almost immediately bring it into disrepute. And so it happens that today the testicular extracts and preparations are probably less used than those of any other organ or portion of the body. This may be the fault of their preparation, and may also be the result of the method of their introduction to the profession.

Much more scientific value seems to pertain to ovarian extracts, which were introduced about three years ago. One of the famous manufacturers puts these preparations on the market in three different forms; an extract of the entire ovary, an extract made from the cortex and a precipitate made from the contents of the ovarian follicles. They are recommended especially in cases of post-operative amenorrhea where serious disturbance has followed the removal of the ovaries, such as headache, sleeplessness, cardiac disturbance, and the like, and, in fact, in some of these instances of so-called cachexia ovaripriva they appear to have been of considerable value. Even in such rather indirect conditions as chlorosis, irritable bladder, etc., they seem to have exercised a beneficial influence. Most of the ovarian extracts made today are made from the ovaries of calves. Seligman, indeed, has warmly advised the use of these remedies in certain nervous disturbances connected with the climacteric and in rosacea of the face. They have also been lauded in certain cases of exophthalmic goitre in women.

While we have long had in our hands preparations like pepsin, ptyalin and pancreatin, which were supposed to contain peculiar digestive ferments, the preparations of an extract of the entire pancreas, akin to that of the thyroid for instance, has been a theoretical novelty of the present decade. It being well known that many cases of diabetes appear to be due to primary disturbance in the pancreas, H. Mackenzie suggested, in 1893, the use of pancreatic extract, and reported the best of results from its employment. His suggestion was quite generally followed, and McNamara went so far as to suggest the use of raw pancreas as a rectal suppository; while Watson Williams advised the implantation in the tissues of a portion of a fresh gland. He lost one patient three days after such an operation. He ascribed the death in this instance to the fact that the gland was removed from a sheep which had been chloroformed, and whose blood was subsequently altered. But this explanation would seem too far-fetched. In 1895 Grube made careful investigations with alcoholic pancreatic extracts, and claimed as the result of his studies that it did influence the cause of cases of diabetes, but that it was without influence upon the elimination of sugar. As a result of these investigations, as well as of those made by Hugoneng and Doyen among the French, it would appear that the glycolytic effect of pancreatic extracts is uncertain, and that the extract itself appears to be, like iodo-thyrine, a more or less unstable substance. And so, of late, we have heard very little of these materials being used for diabetes.

Preparations from the heart muscle were early suggested, and came into early employment, yet have failed to attract very much favor. About the first to use them was Onimus, who employed heart extract by subcutaneous injection in cases of cardiac failure. Hammond also, in this country, took up the subject, although his employment of preparations of this nature has given very little impetus to their general introduction. It would appear that in some cases, however, cardiac tonus has been increased—even that there has been an increase of red corpuscles as well as of the elimination of urine.

In 1898, Onimus recommended the use of extract from the spinal cord, which he proposed to use in certain central diseases, such as myelitis, bulbar paralysis, diabetes, etc. Two years later came into vogue an extract of brain matter (so-called cerebrin) with which the name of Hammond is in this country connected. Its use by injection was recommended in severe nervous prostration, neurasthenia, migraine, hysteria and epilepsy. Success with its use was also reported in chorea, but the remedy does not seem to have won any lasting place in organo-therapy.

Much more widely and favorably known is extract of bone-marrow, whose use in pernicious anemia and certain allied affections seems now to be quite general and well merited. In certain cases, for instance, of pernicious anemia the number of red corpuscles has been apparently quadrupled by its administration, and the proportion of hemoglobin raised from 25 to 80 per cent.

It was but a natural step from bone-marrow to the spleen as a blood-making preparation, since physiology teaches us that marrow and spleen both have a great deal to do with elaboration of the blood. Splenic extract seems especially indicated in anemia, and chlorosis, as well as in the cachexiæ of such chronic poisoning as malaria, etc.

Adrenal extract is the natural outcome of the fact that the adrenals are apparently intimately concerned in the causation of Addison's disease. My colleague, Dr. Stockton, has cured, I believe, cases of Addison's disease with this preparation. Senator was among the first to observe benefit by prolonged administration of this remedy. Mankowski, and other Russian observers who have been studying the subject for a long time, agree that this particular preparation possesses powerful stimulating properties upon the cardiac and respiratory centers. Most interesting is their observation that by the injection of adrenal extract into the veins death from what would otherwise be lethal amounts of chloroform is prevented. Even one or two cc. of a 1 per cent. solution will bring this about in animals. Powerful as this agent is, in this respect it is, nevertheless, by itself one to be used with great caution, and it is not likely that at present at least, it will come into any general use for this purpose. Fuerth succeeded, after some 2,000 endeavors, in separating the peculiar active ingredient in the adrenal which gives to its extract these peculiar properties. It seems to be contained within the pigmentary portion of the organ. Given internally it increases blood pressure very notably, while externally it has more or less local astringent effect. The astringent effect of these preparations has also been taken advantage of, first in this country by Bates, who recommended the employment of solutions of adrenal extract in ophthalmology. In my own experience I have found them to be possessed of remarkable astringent properties, their effect upon exposed mucous membranes being almost instantaneous.

Aside from the extracts made from suprarenals, we have also those made from the kidneys themselves, which have been used in contracted kidneys, calculous nephritis, etc. Some of these preparations have been .gylcerine extracts; others are made in solid form. Under their use in some cases albumin has disappeared and toxicity has returned to the urine.

Aside from the preparations already spoken of, there have been placed on the market, by various foreign and domestic firms, tablets or extractives made from the liver, from the lymph nodes, the mammary glands, from the parotid gland, from the prostate, the thymus and even from the uterus. There have also been prepared extracts from the pituitary body and the muscular structure of the Fallopian tubes. From the bronchial nodes have been prepared substances called pulmonin and glandulin, the former being made also from the lung substance proper. In fact, the advocates of organo-therapy, like those of other novelties, have gone wild in their enthusiasm and their determination to find in these materials remedies for all diseases. The pendulum has, undoubtedly, swung altogether too far in this direction, but it will gradually return to a position of stability and rest, and when it has reached that point we shall undoubtedly find that in certain of these animal extractives there inhere properties of no small value, of which the judicious physician should willingly avail himself.

